# Screening of major hepatotoxic components of *Tripterygium wilfordii* based on hepatotoxic injury patterns

**DOI:** 10.1186/s12906-023-03836-w

**Published:** 2023-01-10

**Authors:** Meng Li, Qiong Luo, Xi Chen, Furong Qiu, Yanyan Tao, Xin Sun, Chenghai Liu

**Affiliations:** 1 Institute of Liver Diseases, Shuguang Hospital affiliated with Shanghai University of Traditional Chinese Medicine, 528 Zhangheng Road, Pudong New Area, Shanghai, 201203 China; 2 Shanghai Key Laboratory of Traditional Chinese Clinical Medicine, Shanghai, 201203 China; 3 Lab of Clinical Pharmacokinetics, Shuguang Hospital affiliated with Shanghai University of Traditional Chinese Medicine, Shanghai, 201203 China; 4 Key Laboratory of Liver and Kidney Diseases, Ministry of Education, Shanghai, 201203 China; 5 Shanghai Innovation Center of TCM Health Service, Shanghai, 201203 China

**Keywords:** *Tripterygium wilfordii* hook. F., Hepatotoxicity, Drug-induced liver injury, Herbal component, Macrophage

## Abstract

**Background:**

*Tripterygium wilfordii* Hook. F. (TwHF), a traditional Chinese medicine, is widely used in the treatment of rheumatoid arthritis. Due to multiorgan toxicity, particularly hepatotoxicity, the application of TwHF is restricted. To clarify the hepatotoxic substances, zebrafish, hepatocytes and macrophages were used for screening based on hepatotoxic injury patterns. This study provides a basis for further elucidation of the hepatotoxic mechanism of TwHF.

**Methods:**

First, 12 compounds were selected according to the chemical categories of TwHF. The fluorescence area and fluorescence intensity of zebrafish livers were observed and calculated. The viability of two hepatocyte lines was detected by CCK_8_ assay. TNF-α and IL-1β mRNA expression in bone marrow-derived macrophages was used to evaluate macrophage activation, a factor of potential indirect hepatotoxicity. Finally, the hepatotoxic characteristics of 4 representative components were verified in mice in vivo.

**Results:**

Parthenolide, triptolide, triptonide, triptobenzene H, celastrol, demethylzeylasteral, wilforlide A, triptotriterpenic acid A and regelidine significantly reduced the fluorescence area and fluorescence intensity of zebrafish livers. The viability of L-02 or AML-12 cells was significantly inhibited by parthenolide, triptolide, triptonide, celastrol, demethylzeylasteral, and triptotriterpenic acid A. Parthenolide, triptolide, triptonide, celastrol, demethylzeylasteral and triptobenzene H significantly increased TNF-α and IL-1β mRNA levels in macrophages, while triptophenolide, hypodiolide and wilforine significantly reduced TNF-α and IL-1β mRNA levels. Triptotriterpenic acid A, celastrol and triptobenzene H at a dose of 10 mg/kg significantly increased the levels of mouse serum alanine aminotransferase and aspartate aminotransferase and aggravated liver inflammation.

**Conclusions:**

Parthenolide, triptolide, triptonide, celastrol, demethylzeylasteral, triptotriterpenic acid A and triptobenzene H might be the main hepatotoxic components of TwFH. Among them, only triptotriterpenic acid A presents direct hepatotoxicity. Triptobenzene H exerts indirect liver damage by activating macrophages. Parthenolide, triptolide, triptonide, celastrol, and demethylzeylasteral can directly and indirectly cause liver injury.

## Introduction

*Tripterygium wilfordii* Hook. F. (TwHF) is a member of the Celastraceae family of perennial vine-like plants. It is a well-known herbal medicine with potential anti-inflammatory and immunomodulatory properties and is widely used for various autoimmune-mediated inflammatory diseases, including rheumatoid arthritis, systemic lupus erythematosus, psoriasis, pemphigus, and Behcet’s disease [1–3]. *Tripterygium wilfordii* polyglycoside tablet, a kind of Tripterygium wilfordii preparation, was once recognized by the World Health Organization as “China’s first new plant drug preparation” for the treatment of arthritis [4]. Thus, the therapeutic value of TwHF was widely recognized. However, there are increasing reports about its side effects, such as reproductive toxicity, hepatotoxicity, nephrotoxicity, cardiotoxicity and splenotoxicity [5]. Due to these adverse effects, the clinical use of TwHF-related drugs is limited [6].

Hepatotoxicity is a main characteristic examined in drug safety evaluation and an important reason for drug withdrawal after marketing. TwHF can cause multiple types of organ injury, among which hepatotoxicity has the highest incidence at up to 40% [7]. TwHF is known to contain more than 200 chemical components, such as triptolide, triptonide, triptobenzene H, celastrol, wilforlide A, and triptotriterpenic acid A. The alkaloids, diterpenoids and triterpenoids in TwHF have varying degrees of toxicity [8, 9]. Triptolide (TPL) is a hepatotoxic substance that can directly injure hepatocytes through mitochondrial depolarization [10, 11], but some herbal or synthetic products containing much lower TPL also cause liver injury. This indicates that there are many hepatic toxins and mechanisms yet to be determined. Differ from direct or idiosyncratic liver injury, a number of experts and scholars recently had proposed a “new pattern”: indirect liver injury, which was medication’s actions rather than its inherent hepatotoxic effects or immunogenicity induced liver injury [12]. As TwHF is an immunomodulator, more new indirect liver injury mechanisms of TwHF need to be further explored. The identification and elucidation of the toxic components and mechanism will be helpful for the rational clinical application of TwHF preparations.

In the current study, we aimed to evaluate the hepatotoxicity of different components in TwHF and their main mechanisms. According to the chemical categories and concentrations of components in TwHF, we performed zebrafish-based toxicity screening of 12 compounds. Furthermore, we explored the types of hepatotoxic damage in two cell lines and validated the representative components in mice. This work contributes to a better understanding of hepatotoxic substances and possible targeted cytological characteristics in liver injury caused by TwHF and lays a foundation for further research on the toxicological mechanism.

## Materials and methods

### Drugs

Parthenolide, triptolide, triptonide, triptophenolide, triptobenzene H, hypodiolide, celastrol, demethylzeylasteral, wilforlide A, triptotriterpenic acid A, wilforine, and regelidine (ACMEC Biochemical, Shanghai, Table 1) were dissolved in DMSO at a concentration of 50 mM and stored at − 20 °C.

**Table 1:** The 12 chemical components of TwHF

Components		Chemical formula	No./CAS	Product code	Purity (%)
Diterpenes	Triptolide	C_20_H_24_O_6_	38,748–32-2	T54611	98
	Triptonide	C_20_H_22_O_6_	38,647–11-9	T42921	98
	Triptophenolide	C_20_H_24_O_3_	74,285–86-2	T67461	98
	Triptobenzene H	C_21_H_28_O_4_	146,900–55-2	T16850	97
	Hypodiolide	C_20_H_30_O_3_	139,122–81-9	T84750	90
Triterpenes	Celastrol	C_29_H_38_O_4_	34,157–83-0	C69551	98
	Demethylzeylasteral	C_29_H_36_O_6_	107,316–88-1	D17480	98
	Wilforlide A	C_30_H_46_O_3_	84,104–71-2	W52141	98
	Triptotriterpenic acid A	C_30_H_48_O_4_	84,108–17-8	T96110	97
Alkaloids	Wilforine	C_43_H_49_NO_18_	11,088–09-8	W36430	98
	Regelidine	C_35_H_37_NO_8_	114,542–54-0	R82510	95
Sesquiterpene	Parthenolide	C_15_H_20_O_3_	20,554–84-1	P15551	98

### Drug toxicology screening in zebrafish

The transgenic liver-fluorescent zebrafish line Tg(L-FABP: EGFP) was used for drug toxicology screening [13]. Zebrafish were allowed to develop for 72 hours post fertilization, and young zebrafish with normal development were selected and transferred to a 24-well plate at a density of 10 juveniles per well. According to the concentration range of the preliminary experiment, 12 components were divided into roughly 4 groups according to the IC50 of each component to liver cell line AML-12 in vitro. All the drugs in this group used the same concentration gradient. A blank control group and three concentration groups were set with 2 replicate wells in each group. Parthenolide, triptophenolide, triptobenzene H, hypodiolide, wilforlide A, triptotriterpenic acid A, regelidine and wilforine were set to the concentration gradient of 7.5, 15, 30 μM, celastrol and demethylzeylasteral were set to the concentration gradient of 0.625, 1.25, 2.5 μM. Triptonide was set to the concentration gradient of 12.5, 25, 50 nM. Triptolide were set to the concentration gradient of 7.5, 15, 30 nM (Table 3). Then, the zebrafish larvae were incubated at a constant temperature (28 °C) in a light incubator, and the drugs were administered for 3 consecutive days. The death and deformity of zebrafish were observed and recorded within 24, 48, and 72 hours after incubation. After 72 hours, the zebrafish larvae were anesthetized with tricaine (0.3‰) and fixed on a glass slide with methylcellulose (4%) for imaging. The fluorescence area and intensity in the liver were calculated. A decrease in fluorescence indicates toxicity.

### Cell culture

According to the L-02 (human liver cell line) and AML-12 (mouse liver cell line) cell growth curves, the drug incubation time to evaluate the dose-effect relationship was selected as 24 hours. L-02 cells were obtained from the ATCC (CRL-12461) and maintained in 5% CO_2_ at 37 °C in RPMI-1640 medium supplemented with 10% fetal bovine serum (FBS, Gibco, Grand Island, NY), 100 U/mL penicillin, and 100 μg/mL streptomycin (Gibco, Gaithersburg, MD). AML-12 cells were obtained from the ATCC (CRL-2254) and grown in DMEM/F-12 supplemented with 10% fetal bovine serum, 100 U/mL penicillin, 100 μg/mL streptomycin, 1× insulin-transferrin-selenium (ITS) mixture, and 40 ng/mL dexamethasone. The cells were maintained at 37 °C in an atmosphere of 5% CO_2_. Bone marrow-derived macrophages (BMDMs) were flushed from the tibias and femurs of 8- to 12-week-old C57BL/6 mice. The cells were cultured in DMEM (Gibco) supplemented with 10% FBS and 1% penicillin–streptomycin (Gibco), differentiated with 20% L929 supernatant for 7 days and maintained in 5% CO_2_ at 37 °C. The BMDMs were dispensed into 6-well culture plates at a concentration of 5 × 10^5^ cells/well and stimulated with the 12 components for 24 h.

### Cell viability assay

L-02 and AML-12 cells were seeded in 96-well culture plates at a concentration of 1 × 10^4^ cells/well and incubated at 37 °C for 24 h in a culture incubator. Then, different concentrations of 12 components (< 1‰ DMSO preparation) were added, and the cells were incubated for another 24 h. 2 ~ 3 concentrations among 6.25 nM, 12.5 nM, 25 nM, 50 nM, 100 nM for triptolide, triptonide, demethylzeylasteral and celastrol, or 1.5625 μM, 3.125 μM, 6.25 μM, 12.5 μM, 25 μM for parthenolide, triptobenzene H, wilforlide A, regelidine, triptophenolide, hypodiolide, wilforine and triptotriterpenic acid A. AML-12 cells were incubated with drug-containing or drug-free BMDM cells supernatants for 24 hours. A total of 100 μl of Cell Counting Kit-8 (CCK-8) solution (Dojindo Biotechnology, Kumamotoi, Japan) homogenized in medium was added to each well, and the plates were incubated for 2 h at 37 °C. The absorbance at 450 nm was measured using a microplate reader.

### RT–PCR

Total RNA was extracted from BMDMs using a Nucleospin RNA II Kit (Takara, Japan). Quantitative RT–PCR was performed with SYBR Green (Applied Biosystems, Foster City, CA, USA). For the thermal cycling procedure, an initial denaturation and activation of the hot-start Taq polymerase at 95 °C for 30 s was followed by 40 cycles consisting of denaturation (95 °C for 5 s) and annealing and elongation (60 °C for 34 s). Finally, a dissociation curve step was added (95 °C for 15 s, 60 °C for 1 min, 95 °C for 15 s). See Table 2 for the detailed primer sequences.

**Table 2:** Primer sequences

Gene	Sequences (5′-3′)	
	Forward primer	Reverse primer
TNF-α	TAG CCA GGA GGG AGA ACA GA	CCA GTG AGT GAA AGG GAC AGA
IL-1β	TAC ATC AGC ACC TCA CAA GC	AGA AAC AGT CCA GCC CAT ACT
IL-10	AGT GTG TAT TGA GTC TGC TGG	GAG AGA GGT ACA AAC GAG GTT
β-actin	TGGAATCCTGTGGCATCCATGAAAC	TAAAACGCAGCTCAGTAACAGTCCG

### Mouse acute liver toxicity test

Five representative components were selected for further liver toxicity detection in vivo to confirm the reliability of the hepatotoxicity findings. Wild-type C57BL/6 mice (male, 8 weeks old) were obtained from the Shanghai Lab Animal Research Center (Shanghai, China) and randomly divided into 11 groups of 6 mice. While the blank control group was given an equal dose of 5% DMSO, the other groups were treated with different doses of triptotriterpenic acid A (1 mg/kg and 10 mg/kg, dissolved in 5% DMSO, i.p.), celastrol (1 mg/kg and 10 mg/kg, dissolved in 5% DMSO, i.p.), triptobenzene H (1 mg/kg and 10 mg/kg, dissolved in 5% DMSO, i.p.), wilforlide A (1 mg/kg and 10 mg/kg, dissolved in 5% DMSO, i.p.), and wilforine (1 mg/kg and 10 mg/kg, dissolved in 5% DMSO, i.p.). The mice were sacrificed 24 hours after acute administration, and plasma and liver tissue samples were collected for further detection.

### Determination of serum ALT and AST

Blood was collected, and serum was separated by centrifugation (4 °C, 3000 rpm, 15 min). The levels of serum ALT and AST were determined according to the requirements of the instructions provided in reagent kits (Nanjing Jiancheng Institute of Biological Engineering, Cat No. C009–1 and Cat No. A059–1).

### HE staining of liver tissues

The liver tissues of mice were fixed in 10% neutral phosphate-buffered formalin solution and then dehydrated and embedded in paraffin to make conventional paraffin sections. The sections were cut into 4 μm thick sections and stained with hematoxylin and eosin (H&E, Nanjing Jiancheng Institute of Biological Engineering, Cat No. D006). The histopathological changes in liver tissues were observed under an optical microscope.

### Statistical analysis

All values are presented as the means ± standard deviations (SDs). The data were analyzed using analysis of variance (ANOVA) with *Dunnett’s* post-hoc test for multiple comparisons or Student’s *t* test (normal distributed data) for two groups. *P* values < 0.05 were considered to indicate statistical significance.

## Results

### Nine components of TwHF were hepatotoxic to zebrafish

**Fig. 1:**
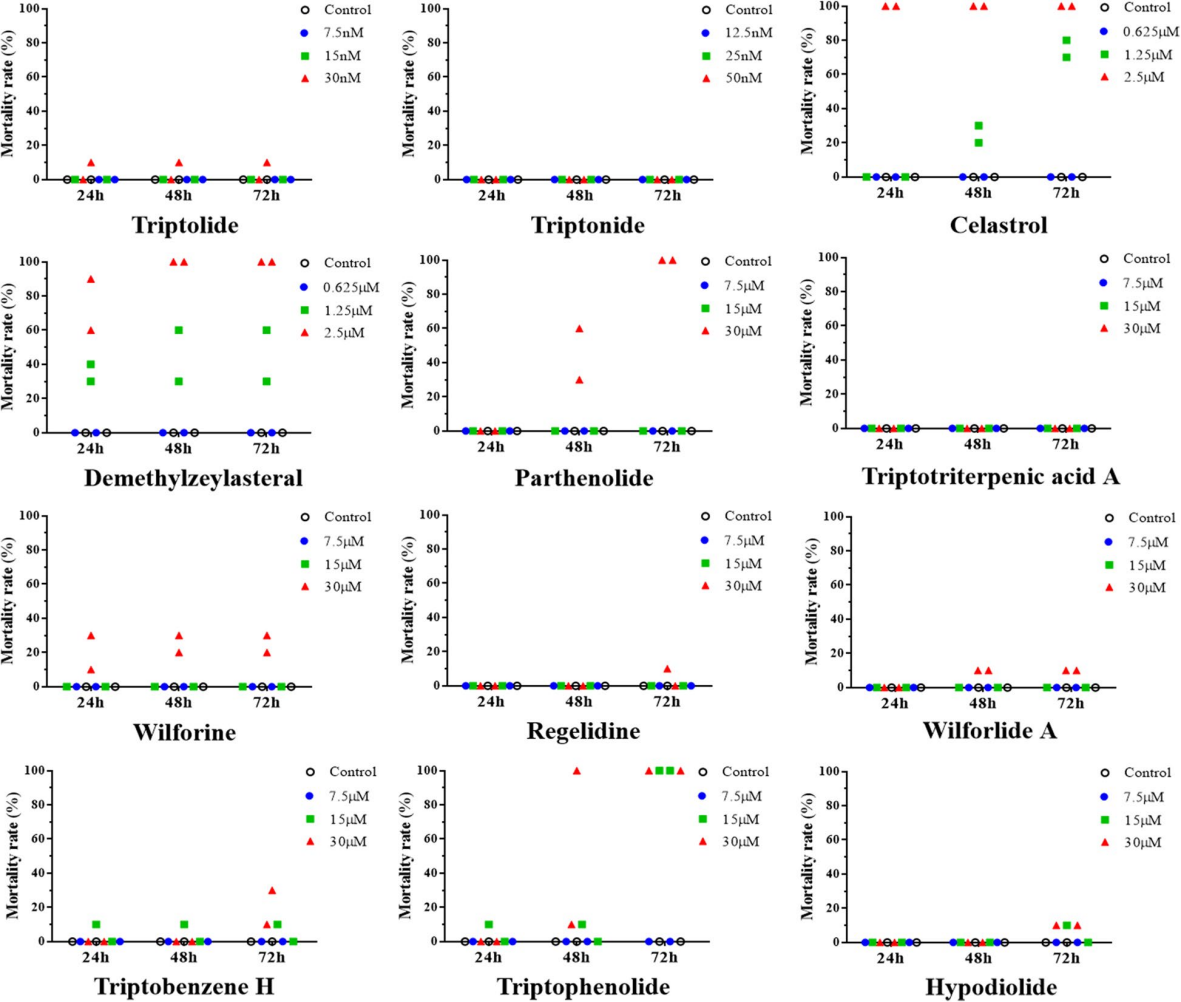
Effects of the 12 components on the mortality rate of zebrafish. Notes: *N* = 10 per well; 2 replicate wells was set in each group

**Fig. 2:**
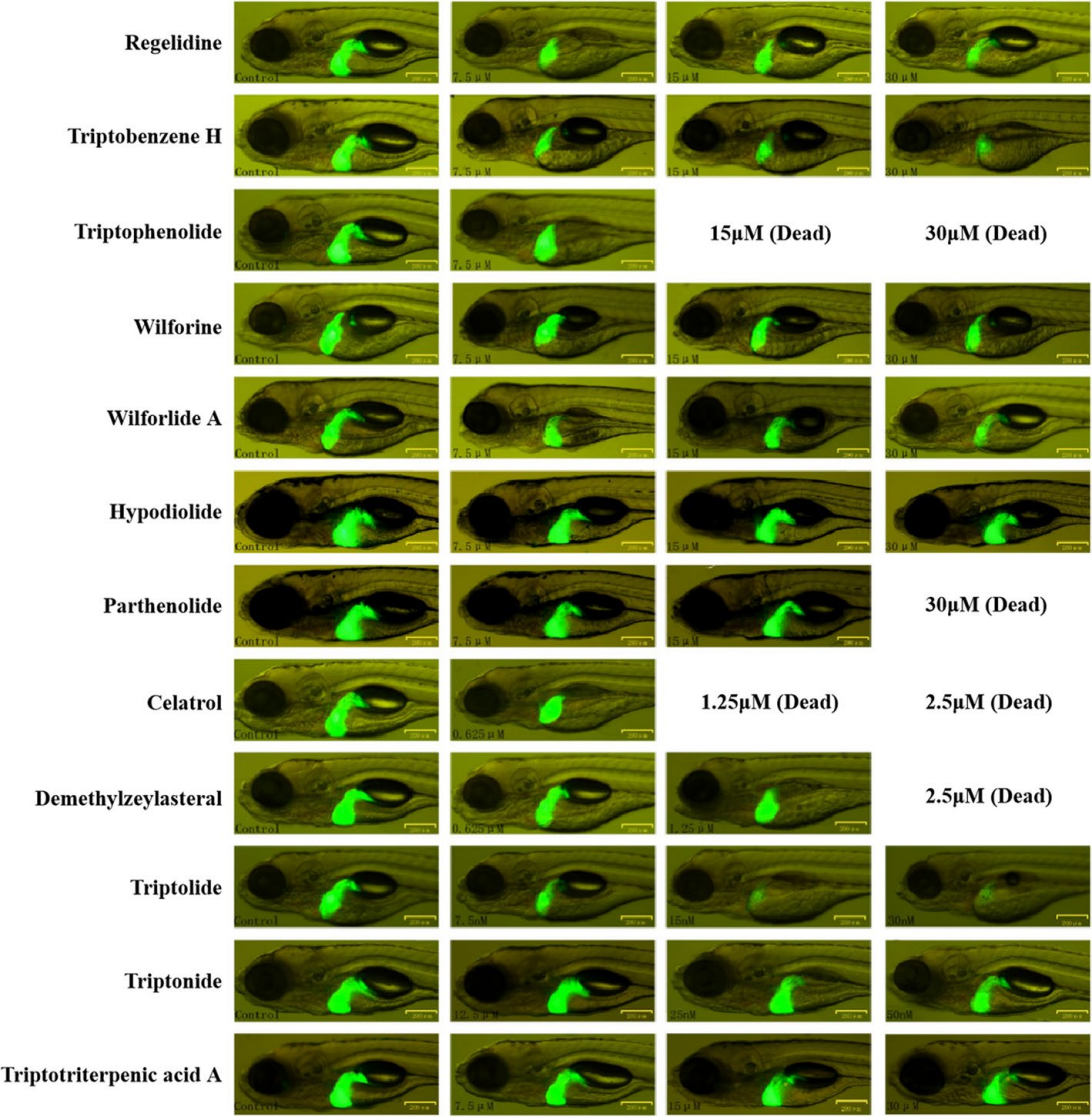
Fluorescence images of zebrafish livers. Notes: after 72 h of drug intervention; *N* = 10; scale bar = 200 μm

First, celastrol (1.25 μM for ≥48 h, 2.5 μM for ≥24 h), demethylzeylasteral (1.25 μM or 2.5 μM for ≥24 h), parthenolide (30 μM for ≥48 h), wilforine (30 μM for ≥24 h), wilforlide A (30 μM for ≥48 h), Triptobenzene H (15 μM or 30 μM for ≥72 h), triptophenolide (15 μM or 30 μM for ≥48 h, not yet determined) and hypodiolide (15 μM for ≥72 h) were observed to impact the mortality of zebrafish (Fig. 1). The fluorescence area and fluorescence intensity in the zebrafish liver were calculated, and hepatotoxicity was evaluated after 72 h of drug intervention. The results showed that parthenolide, triptolide, triptonide, triptobenzene H, celastrol, demethylzeylasteral, wilforlide A, triptotriterpenic acid A and regelidine significantly reduced the fluorescence area and intensity in the zebrafish liver (Fig. 2, Table 3). Within the concentration range we observed, triptophenolide, hypodiolide and wilforine had no significant hepatotoxic effects on zebrafish. However, we were not able to investigate all concentration levels of triptophenolide, hypodiolide and wilforine due to mortality.

**Table 3:** Fluorescence area and fluorescence intensity in zebrafish livers

Group		Fluorescence intensity (mean ± SD, %)	Fluorescence Area (mean ± SD, %)	Group		Fluorescence intensity (mean ± SD, %)	Fluorescence area (mean ± SD, %)
Parthenolide	Control	100.00 ± 5.22	100.00 ± 0.89	Celastrol	Control	100.00 ± 0.59	100.00 ± 4.07
	7.5 μM	70.46 ± 0.26*	98.21 ± 1.09		0.625 μM	75.61 ± 1.43**	63.89 ± 0.38*
	15 μM	78.61 ± 1.94	90.65 ± 1.70*		1.25 μM	(Dead)	(Dead)
	30 μM	(Dead)	(Dead)		2.5 μM	(Dead)	(Dead)
Triptolide	Control	100.00 ± 0.35	100.00 ± 3.50	Demethylzeylasteral	Control	100.00 ± 0.59	100.00 ± 4.07
	7.5 nM	65.14 ± 2.36**	86.63 ± 0.46		0.625 μM	89.41 ± 3.19	86.49 ± 0.23
	15 nM	57.50 ± 3.44**	77.97 ± 1.50*		1.25 μM	72.48 ± 0.28**	77.04 ± 0.15*
	30 nM	41.42 ± 3.09**	67.35 ± 4.48*		2.5 μM	(Dead)	(Dead)
Triptonide	Control	100.00 ± 0.59	100.00 ± 4.07	Wilforlide A	Control	100.00 ± 3.61	10.00 ± 1.35
	12.5 nM	76.05 ± 2.21**	92.20 ± 4.79		7.5 μM	62.68 ± 0.19**	84.68 ± 0.33**
	25 nM	77.24 ± 2.25*	87.90 ± 2.22		15 μM	41.51 ± 1.95**	60.34 ± 4.65*
	50 nM	58.31 ± 5.71*	80.95 ± 2.89		30 μM	41.28 ± 0.93**	62.65 ± 4.13*
Triptophenolide	Control	100.00 ± 0.35	100.00 ± 3.50	Triptotriterpenic acid A	Control	100.00 ± 0.83	100.00 ± 0.99
	7.5 μM	109.71 ± 0.99	95.82 ± 1.81		7.5 μM	84.50 ± 1.86*	91.81 ± 0.08*
	15 μM	(Dead)	(Dead)		15 μM	80.48 ± 3.26*	84.76 ± 1.57*
	30 μM	(Dead)	(Dead)		30 μM	83.87 ± 2.47*	87.07 ± 0.49**
Triptobenzene H	Control	100.00 ± 3.61	100.00 ± 1.35	Regelidine	Control	100.00 ± 0.35	100.00 ± 3.50
	7.5 μM	63.47 ± 2.88*	73.38 ± 2.39*		7.5 μM	67.22 ± 1.71**	84.22 ± 1.29
	15 μM	41.57 ± 1.42**	61.04 ± 2.35**		15 μM	56.00 ± 0.25**	87.71 ± 3.29
	30 μM	29.57 ± 3.75**	50.75 ± 1.48**		30 μM	51.36 ± 1.58**	82.11 ± 0.57*
Hypodiolide	Control	100.00 ± 55.22	100.00 ± 3.61	Wilforine	Control	100.00 ± 1.35*	100.00 ± 3.61
	7.5 μM	90.68 ± 5.78	93.62 ± 3.95		7.5 μM	78.55 ± 2.35	81.41 ± 5.90
	15 μM	95.26 ± 4.42	97.55 ± 1.30		15 μM	76.17 ± 2.62*	78.45 ± 7.02
	30 μM	89.99 ± 1.80	89.59 ± 1.37*		30 μM	79.20 ± 1.98*	80.09 ± 6.30

*N* = 10

^*^P < 0.05

^**^P < 0.01 vs. the control group

### Six components of TwHF were directly toxic to hepatocytes (L-02 and AML-12 cell lines)

To screen out components of TwHF with direct hepatotoxicity, two parenchymal hepatic cell lines, L-02 and AML-12, were used for evaluation. Cell viability was detected with a CCK-8 kit, and the 50% inhibitory concentration (IC_50_) values were calculated. Parthenolide, triptolide, triptonide, celastrol and demethylzeylasteral showed significant inhibitory effects on hepatocyte activity (Table 4). Although triptotriterpenic acid A had no obvious toxicity against L-02 cell line, its IC50 against AML-12 cell line was 16.09 ± 1.32 μM. Therefore, triptotriterpenic acid A was also considered to have suspicious hepatotoxicity. The other six components of TwHF did not exhibit apparent toxicity to either L-02 or AML-12.

**Table 4:** IC_50_ values of the 12 components in the L-02 and AML-12 cell lines

Component	50% inhibitory concentration (IC_50_) value	
	L-02 cell line	AML-12 cell line
Parthenolide	(11.47 ± 1.03) μM	NA
Triptolide	(28.06 ± 1.02) nM	(27.77 ± 1.09) nM
Triptonide	(75.79 ± 8.88) nM	(91.49 ± 3.10) nM
Triptophenolide	NA	NA
Triptobenzene H	NA	NA
Hypodiolide	NA	NA
Celastrol	(6.74 ± 1.18) μM	(4.62 ± 1.30) μM
Demethylzeylasteral	(2.02 ± 1.03) μM	(4.03 ± 1.78) μM
Wilforlide A	NA	NA
Triptotriterpenic acid A	NA	(16.09 ± 1.32) μM
Regelidine	NA	NA
Wilforine	NA	NA

*N* = 6

*NA* Not Available; the highest concentration tested was 25 μM; three experimental replicates in all panels

### Six components of TwHF activated mouse macrophages (BMDMs)

**Fig. 3:**
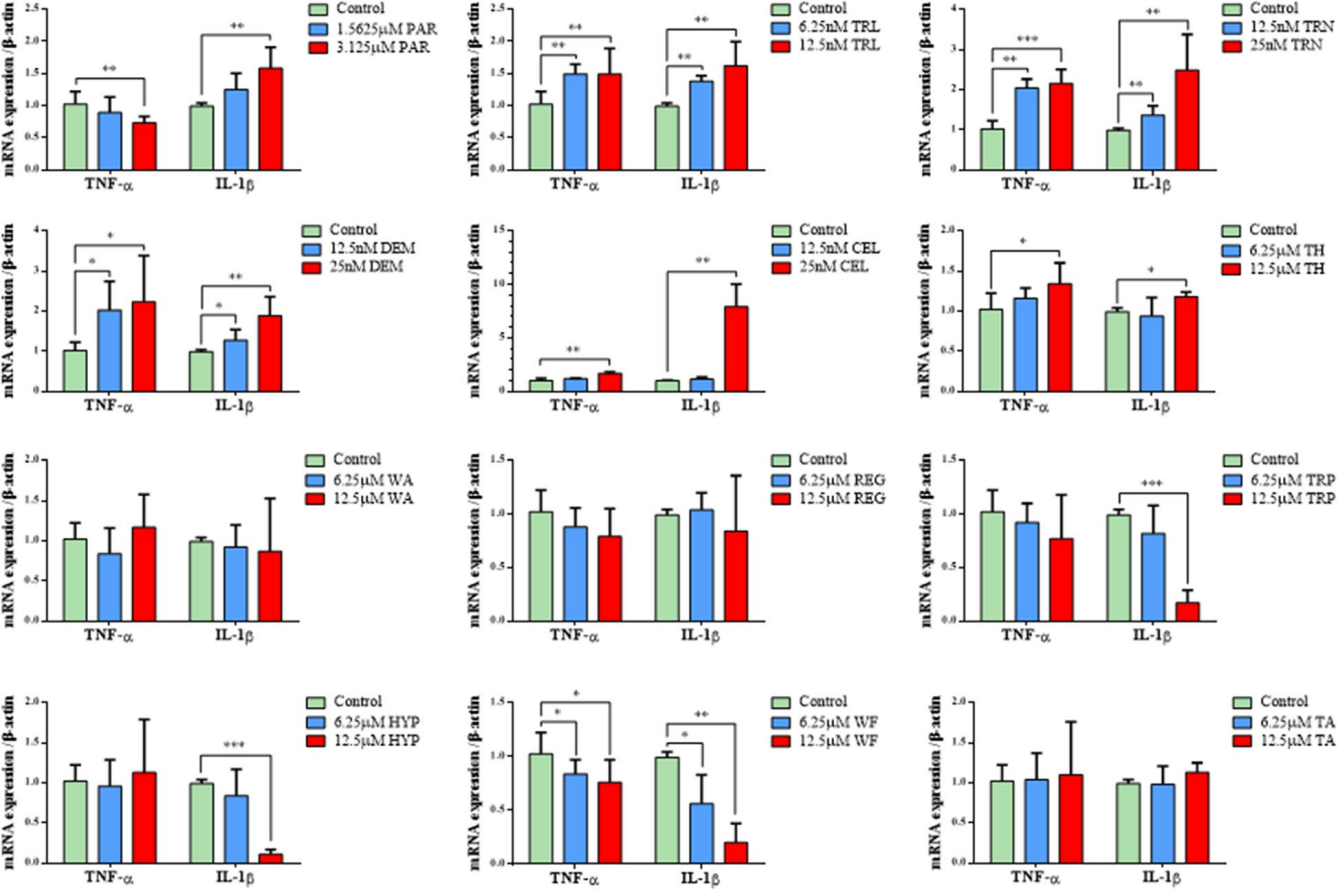
Effects of 12 components of TwHF on the mRNA expression levels of TNF-α and IL-1β. Notes: *N* = 6; *P < 0.05, **P < 0.01, ***P < 0.001 vs. the control group; three experimental replicates in all panels. PAR, parthenolide; TRL, triptolide;TRN, triptonide; DEM, demethylzeylasteral; CEL, celastrol; TH, triptobenzene H; WA, wilforlide A; REG, regelidine; TRP, triptophenolide; HYP, hypodiolide; WF, wilforine; TA, triptotriterpenic acid A

Immune-mediated indirect hepatotoxicity is emerging as a liver injury pattern [14]. A large number of studies have confirmed that TNF-α, IL-1β, IL-6 and other proinflammatory cytokines secreted by macrophages can aggravate liver injury and promote hepatocyte apoptosis [15]. Thus, the mRNA expression levels of TNF-α and IL-1β in macrophages were detected by RT–PCR after incubation with each component of TwHF. The results showed that parthenolide, triptolide, triptonide, celastrol, demethylzeylasteral and triptobenzene H significantly increased the mRNA expression levels of TNF-α and IL-1β. In contrast, wilforine significantly reduced the mRNA expression levels of TNF-α and IL-1β. Triptophenolide and Hypodiolide only reduced the mRNA expression of IL-1β, but not TNF-α. However, wilforlide A, regelidine and triptotriterpenic acid A did not significantly affect the mRNA expression of TNF-α and IL-1β (Fig. 3).

### Celastrol and Triptobenzene H indirectly injury hepatocyte through activation of macrophages

**Fig. 4:**
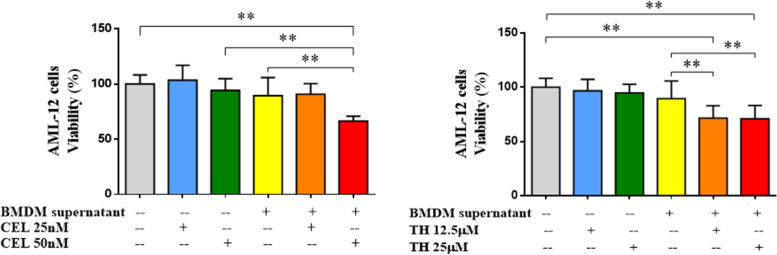
Effect of BMDM supernatant preincubated with CEL or TH on AML-12 cells. Notes: *N* = 6; **P < 0.01; three experimental replicates in all panels. CEL, celastrol; TH, triptobenzene H

Next, AML-12 cell lines were treated with BMDM supernatant preincubated with celastrol or triptobenzene H for 24 hours, respectively. The results of CCK-8 showed that the BMDM supernatant preincubated with 50 nM celastrol, 12.5 μM or 25 μM triptobenzene H significantly inhibited the viability of AML-12 cells, while BMDM supernatant alone had no effect on AML-12 cells viability. The viability of AML-12 cells was not significantly inhibited after only using different concentrations of celastrol or triptobenzene H for 24 hours. Therefore, celastrol or triptobenzene H may have an indirect effect on liver injury by activating macrophages (Fig. 4).

### Acute hepatotoxicity validation of 5 representative components from TwHF in mice

**Fig. 5:**
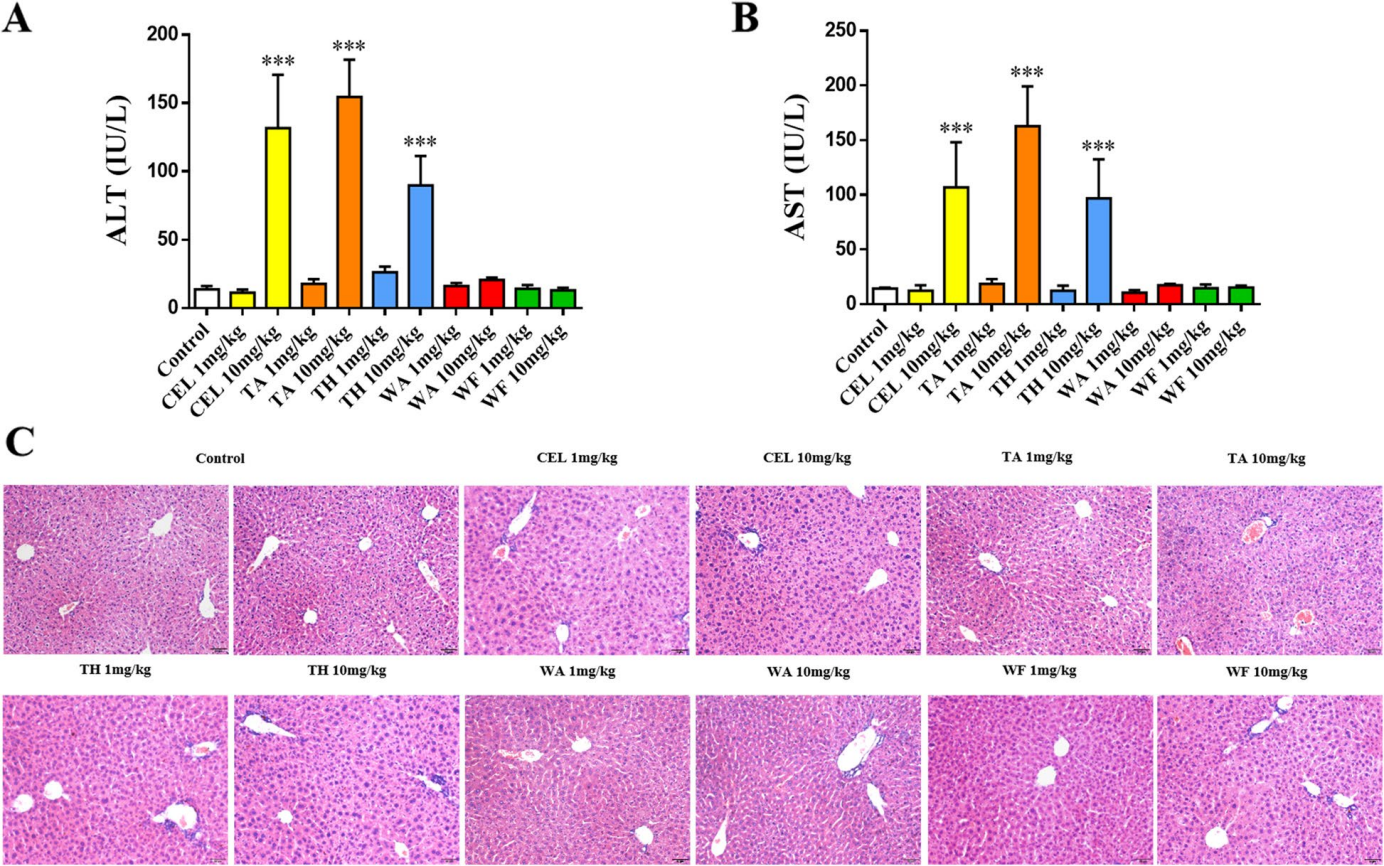
Acute hepatotoxicity validation of 5 representative components of TwHF in mice. Notes: **A** Serum ALT levels in mice; **B** Serum AST levels in mice; **C** HE staining images of mouse liver tissue slices, 200×; *N ≥* 6; ***P < 0.001 vs. the control group. CEL, celastrol; TA, triptotriterpenic acid A; TH, triptobenzene H; WA, wilforlide A; WF, wilforine

To verify whether these components have acute hepatotoxicity in mammals, we selected 5 representative components for further observation according to the results above. C57BL/6 mice were intraperitoneally injected with celastrol (CEL), triptotriterpenic acid A (TA), triptobenzene H (TH), wilforlide A (WA) and wilforine (WF). After 24 hours of administration, serum ALT and AST levels were determined, and liver tissue slices were stained with HE. The results showed that serum ALT and AST were significantly increased in the groups treated with CEL, TA and TH and 10 mg/kg, while the values in the other groups were normal (Fig. 5A and B). In HE-stained pathological images, these 3 groups exhibited slightly disordered liver structures and mild swelling of hepatocytes with scattered inflammatory cell infiltration to varying degrees. The slices from the normal control group and the other groups showed intact hepatic lobules, neatly arranged hepatic cords, and no obvious infiltration of inflammatory cells (Fig. 5C).

## Discussion

Drug-induced liver injury (DILI) has gradually become an important aspect of drug safety evaluation. The list of herbal products causing liver injury has expanded in recent years [16]. It has been reported that 23% of DILI cases are caused by Chinese herbal medicines, and approximately 20% of hospitalized patients with acute liver injury have herb-induced liver injury (HILI) [17]. In China, TwHF ranks first in terms of the liver injury-causing single traditional Chinese medicines (TCMs), and its clinical application and promotion have been greatly affected. Therefore, the hepatotoxic components and mechanisms of TwHF have gradually become popular research topics. Triptolide, one of the main active components of TwFH, is also one of the main hepatotoxic factors related to membrane damage, mitochondrial destruction, metabolic dysfunction, endoplasmic reticulum stress, oxidative stress, apoptosis and autophagy [18]. Previous researchers have found that triptolide, celastrol and demethylzeylasteral are the main hepatotoxic components of TwHF based on spectrum-effect correlation analysis [19]. Our study found that in addition to these three components, other components in TwHF also have hepatotoxicity. Moreover, the exploration of the toxic mechanisms revealed that some components have no direct hepatotoxicity. These results suggest that TwHF may have other unknown hepatotoxic mechanisms.

Typically, DILI is classified as either intrinsic or idiosyncratic. Recently, it was recognized that indirect hepatotoxicity is a third type, but in-depth understanding is lacking [20]. This type of DILI usually occurs in the specific population with underlying diseases or susceptibility and manifests when the drug action changes a body state (such as immune homeostasis), thereby inducing liver injury or aggravating the original liver disease. Indirect hepatotoxicity is much more common than idiosyncratic hepatotoxicity; it is a total response to a whole class of drugs, such as tumor necrosis factor antagonists and checkpoint inhibitors, rather than just a specific drug such as nitrofurantoin. It may exist in most HILI cases, but there is a lack of research on this topic [21]. This study may provide new ideas for clarifying the mechanism of HILI.

As well-recognized biological model organisms for toxicology research, zebrafish have become “workhorse” model organisms in chemical toxicity screening [22]. First, by comparing the mortality rate and changes in the liver fluorescence areas of zebrafish, we found that 9 components were obviously hepatotoxic. This result indicated that there were indeed other substances causing liver injury in TwHF besides TP. According to the mechanisms of DILI, the hepatotoxicity was classified as direct and indirect hepatotoxicity. To avoid species differences as much as possible, we adopted two kinds of liver parenchymal cell lines from different species, the L-02 cell line (humans) and the AML-12 cell line (mice). Our results showed that parthenolide, triptolide, triptonide, celastrol, demethylzeylasteral, and triptotriterpenic acid A had direct hepatocellular toxicity, and the toxicity test results for the two cell lines were consistent. These six components might be the basis of the hepatotoxicity of TwHF. Combined with the toxicity results of zebrafish, these results suggest that 3 components that did not show direct hepatocyte toxicity, triptobenzene H, regelidine and wilforlide A, might have indirect hepatotoxicity.

Macrophages, which are widely distributed throughout the body, participate in innate and adaptive immune responses in many diseases. The physiologic functions of macrophages can vary tremendously depending on the environment in which the macrophages reside and the local stimuli to which they are exposed [23]. Activated macrophages secrete proinflammatory cytokines, such as TNF-α and IL-1β, which cause an immune inflammatory response in the liver. Chemokines such as MCP-1 and CXCL-10 can also be secreted to recruit peripheral macrophages and immune cells into the liver and aggravate inflammation [24]. Therefore, macrophage activation was also evaluated after drug treatment in addition to direct hepatocyte toxicity in our study. The TNF-α and IL-1β mRNA expression levels in BMDMs were detected to determine whether macrophages were activated. We found that parthenolide, triptolide, triptonide, celastrol, demethylzeylasteral and triptobenzene H significantly increased the mRNA expression levels of TNF-α and IL-1β. This result suggests that there are components in TwHF that can activate macrophages; thus, macrophage activation may be one of the mechanisms of indirect hepatotoxicity. We further classified the 12 components of TwHF into 5 groups according to the screening results of zebrafish and cell experiments: the hepatocyte-toxicity-only group, the macrophages (Mac)-activation-only group, both the hepatocyte-toxicity-and-Mac-activation group, the non-hepatocyte-toxicity-or-Mac-activation group, and the no-zebrafish-hepatotoxicity group. Finally, 5 representative components from each group were selected for validation in mice, and the results were consistent with those of the cell experiment above. A series of studies has demonstrated that TP induces significant liver damage in mice through a variety of immune pathways, such as activation of NKT cells [25], recruitment of macrophages [26], infiltration of neutrophils [27] and disturbance of the Th17/Treg balance [28]. These results suggest that indirect liver injury is common in DILI and that the culprit of liver injury caused by TwHF cannot only be attributed to its direct toxicity; rather, indirect toxicity is also extremely important.

Of course, there were limitations in our study. Although we compared and analyzed the toxicity of 12 components of TwHF at different model levels, we did not study all the components in TwHF. In the evaluation of liver toxicity in zebrafish, we did not carefully observe the exact hepatotoxicity dose and time of some component. More experiments and dose range need to be further discussed. In addition, only macrophages were considered to explain indirect hepatotoxicity in our study, there were other immune cells involved in indirect hepatotoxicity. Furthermore, influence of TwHF metabolism in vivo on this indirect liver injury was also needed to explore.

## Conclusions

Through evaluation of zebrafish liver injury, hepatocyte viability and macrophage activation, parthenolide, triptolide, triptonide, celastrol, demethylzeylasteral, triptotriterpenic acid A and triptobenzene H were found to be the main hepatotoxic components of TwFH. Among them, only triptotriterpenic acid A presented direct hepatotoxicity. Triptobenzene H produced indirect liver damage by activating macrophages. Parthenolide, triptolide, triptonide, celastrol, and demethylzeylasteral directly and indirectly caused liver injury.

## Data Availability

The datasets generated and/or analyzed during the current study are available from the corresponding author or from https://pan.baidu.com/s/11DUPUroG7UtIANV0ZpfcPQ?pwd=gaja.

## Abbreviations

BMDM: Bone marrow-derived macrophage
CCK-8: Cell counting kit-8
CEL: Celastrol
DEM: Demethylzeylasteral
DILI: Drug-induced liver injury
DMSO: Dimethylsulfoxide
FBS: Fetal bovine serum
HILI: Herb-induced liver injury
HYP: Hypodiolide
IC50: 50% inhibitory concentration
ITS: Insulin-transferrin-selenium
PAR: Parthenolide
RA: Rheumatoid arthritis
REG: Regelidine
TA: Triptotriterpenic acid A
TCM: Traditional Chinese medicine
TH: Triptobenzene H
TPL: Triptolide
TRN: Triptonide
TRP: Triptophenolide
TwHF: *Tripterygium wilfordii* Hook. F.
WA: Wilforlide A
WF: Wilforine
